# Case report: Serotoninergic and cholinergic syndromes induced by self-medication

**DOI:** 10.3389/fphar.2023.1080249

**Published:** 2023-02-16

**Authors:** Sofía Orozco-Solano, Martha Milena Silva-Castro, Manuel Machuca

**Affiliations:** ^1^ Centro de Información de Medicamentos, Servicio de Farmacia, Hospital Dr R. A. Calderón Guardia, San José, Costa Rica; ^2^ Responsable de Investigación de la Sociedad Española de Optimización de la Farmacoterapia, Spain, Spain; ^3^ Universidad Loyola, Dos Hermanas, Sevilla, Spain

**Keywords:** cholinergic syndrome, adverse drug reaction, pharmaceutical care, comprehensive medication management, case report, serotoninergic syndrome

## Abstract

Self-medication is a part of the self-care practices carried out by the elderly in their environment. The aim of this case report is to show how the self-medication of fluoxetine and dimenhydrinate in an older adult can induce serotoninergic and cholinergic syndromes, showing symptoms such as nausea, tachycardia, tremor, loss of appetite, memory loss, decreased vision, falls, and increased urination. An older adult who has been diagnosed with arterial hypertension, dyslipidemia, diabetes mellitus, and a recent diagnosis of essential thrombosis is the subject of this case report. After the analysis of the case, cessation of fluoxetine was recommended to avoid withdrawal symptoms, therefore decreasing the need for dimenhydrinate and the medicines used for dyspepsia. After the recommendation, the patient showed an improvement in the symptoms. Finally, the comprehensive evaluation process of the medication in the Medicines Optimization Unit achieved the detection of the problem and improved the patient’s health condition.

## Introduction

Self-medication is a complex phenomenon in which patients, or their caregivers, decide to administer a medication in the event of discomfort, without prescription. This practice is a part of the self-care model in many areas (Kumar et al., 2022).

Fluoxetine is a selective serotonin reuptake inhibitor used in the treatment of depression (Page et al., 2006). Its most common side effects are nausea and headache (Lexi-Drugs, 2022). Regarding dimenhydrinate, it is an antihistamine with anticholinergic activity as a muscarinic receptor antagonist, prescribed for nausea and vomiting (Page et al., 2006). Among the side effects that it can produce, with undefined frequency, are tachycardia, dizziness, drowsiness, headache, xerostomia, or blurred vision (Lexi-Drugs, 2022).

This case report aims to show how the serotonergic and cholinergic burden affects the elderly, when a high dose of fluoxetine for depression and dimenhydrinate for nausea are used concomitantly, in both cases as self-medication.

## Case report

A 78-year-old male patient with high blood pressure, dyslipidemia, diabetes mellitus, and a recent diagnosis of essential thrombosis (see the summary of assessment of the patient’s drug-related needs in Table 1). He was referred for the first time to the Medicines Optimization Unit of a hospital in Costa Rica, giving his consent in writing for his participation in this case report.

**TABLE 1:** Summary of Assessment of the Patient's Drug-related Needs.

Clinical Condition	Medications	Scheme of prescriptions	Scheme taken by the patient	Observations
Arterial hypertension	Enalapril 20 mg	1-0-1	1-0-0	
	Omeprazole 20 mg	not prescribed	1-0-0	Use if necessary.
	Aluminium hydroxide and magnesium hydroxide	1-1-1	1-1-1	
Acid indigestion	Famotidine 40 mg	0-0-1	0-0-1	Use if necessary.
	Alka-seltzer (anhydrous citric acid, acetylsalicylic acid, sodium bicarbonate)	not prescribed	1-0-0	Use if necessary.
Continuous use as self-medication for nausea	Dimenhydrinate 50 mg	1-1-1	1-1-1-1	Prescription for 10 days, the patient has bought more
Mellitus diabetes	Metformin 500 mg	1-0-1	1-0-0	
Self-medication for antidepressive syndrome	Fluoxetine 20 mg	not prescribed	1-1-1	Sometimes take 2 capsules and in others 3 capsules.
Hypercholesterolemia	Lovastatin 20 mg	0-0-1	1-0-0	Even though he uses the statin in the morning, his cholesterol levels are in the normal range.
Essential thrombosis	Hydroxycarbamide 500 mg	0-2-0	0-1-0	
Not indicated	Diphenhydramine 12.5 mg / 5 mL	5 mL every 8 hours	---	He hasn't taken it

He reported of having recently suffered from nausea, tachycardia, tremor, loss of appetite, memory loss, and a significant decrease in his vision. Also, he had fallen twice and suffers from frequent urination.

On his last visit to the hematologist, he had been ordered to increase the hydroxycarbamide intake to two daily tablets. This was due to the essential thrombosis he presented, and the values that he exhibited in the blood count of April 2022 showed that the hemoglobin, platelets, leukocytes, neutrophils, and lymphocytes were altered (see Table 2). However, due to his current condition, both the patient and his family do not believe that he would be able tolerate the increased dose, and therefore, he has not started the second hydroxycarbamide tablet.

**TABLE 2:** Clinical Laboratory Report, April 2022.

Test name	Result	Flags	Test name	Result	Flags
Glucose	97 mg/dL	Normal	Triglycerides	126.5 mg/dL	Normal
Haemoglobin A_1C_	5.7 %	Normal	Prostate-specific antigen (PSA)	1.37 ng/mL	Normal
Creatinine	0.83 mg/dL	Normal	Haemoglobin	16.4 mg/dL	Normal
Urea nitrogen	11.4 mg/dL	Normal	Platelet	877,000 /mL	High
Cholesterol	121.9 mg/dL	Normal	Leukocytes	17,770 /mL	High
High-density lipoprotein cholesterol (HDL)	41.6 mg/dL	Normal	Neutrophils	14,180 /mL	High
Low-density lipoprotein	55 mg/dL	Normal	Lymphocytes	2,114 /mL	Normal
Very Low-density lipoprotein cholesterol (VLDL)	25.3 mg/dL	Normal			

Due to the anguish caused by having to increase the dose of hydroxycarbamide, the patient and his wife decided to start with fluoxetine. The patient´s wife says that, “our son takes that medication for the nerves,” and both the wife and the daughter report that he no longer leaves the house. Under the wife’s supervision, the patient sometimes takes a 20 mg fluoxetine capsule every 12 or 8 hours, depending on his mood. Additionally, because of the constant nausea during the day, dimenhydrinate was prescribed every 8 hours for 10 days, but the use has been extended continuously and even increasing the dosage to every 6 hours.

Once the assessment of the case was carried out (Cipolle et al., 2012), regarding urinary frequency, the clinical laboratory report (see Table 2) indicated a prostate-specific antigen in the normal result, not related to prostatic hyperplasia; the patient also did not have symptoms such as dysuria, hematuria, or fever that were related to a urinary tract or kidney infection. In addition, from a pharmacovigilance perspective, there is a clear relationship between temporal sequences of the onset of the following symptoms: nausea, tachycardia, tremor, loss of appetite, memory loss, and significant decrease in vision, with the beginning of self-medication with fluoxetine and dimenhydrinate. Given the aforementioned information, the presented symptoms were associated with the presence of serotoninergic and cholinergic syndrome, and it was expected that by gradually decreasing fluoxetine and dimenhydrinate, the symptoms would decrease or even disappear.

The dose of fluoxetine used was observed to produce a high serotonergic burden that manifests with nausea and tachycardia. The high dose of dimenhydrinate used for nausea increases the risk of tachycardia and is also associated with recent memory loss, tremor, blurred vision, and impaired urination. This can be aggravated by the excessive use of drugs that raise the gastric pH, such as omeprazole, which slow down the absorption of drugs, with the risk of increasing their toxicity. For example, fluoxetine can delay its absorption for 1–2 h (Shargel and Yu, 2016). The increase in hydroxycarbamide in this case could also contribute to nausea (Lexi-Drugs, 2022).

A pharmacotherapeutic intervention was aimed to reduce the dosage of fluoxetine, and hence, there was a need for dimenhydrinate and the medications used for stomach problems. Also, to avoid withdrawal syndrome, a process of tapering of both fluoxetine (Horowitz and Taylor, 2022) and dimenhydrinate is proposed. Ever since fluoxetine was started approximately two and a half months ago, as it is an antidepressant with a long half-life (norfluoxetine, an active metabolite with a half-life of approximately 9.3 days) (Lexi-Drugs, 2022), it was recommended to be decreased to one capsule every 2 weeks (Bazire, 2021). Additionally, it was proposed that in case of nausea, only one dimenhydrinate tablet 15 min before lunch be taken (when the patient takes hydroxycarbamide).

In a future follow-up appointment, the withdrawal of lovastatin could be assessed, in case the patient has optimal cholesterol values and lovastatin is taken at an off time, which suggests a doubtful effectiveness. A hypothesis could be that lovastatin is slowing down the absorption of fluoxetine, increasing plasma levels and being co-responsible for the serotoninergic syndrome (Ghanizadeh and Hedayati, 2013).

**FIGURE 1:**
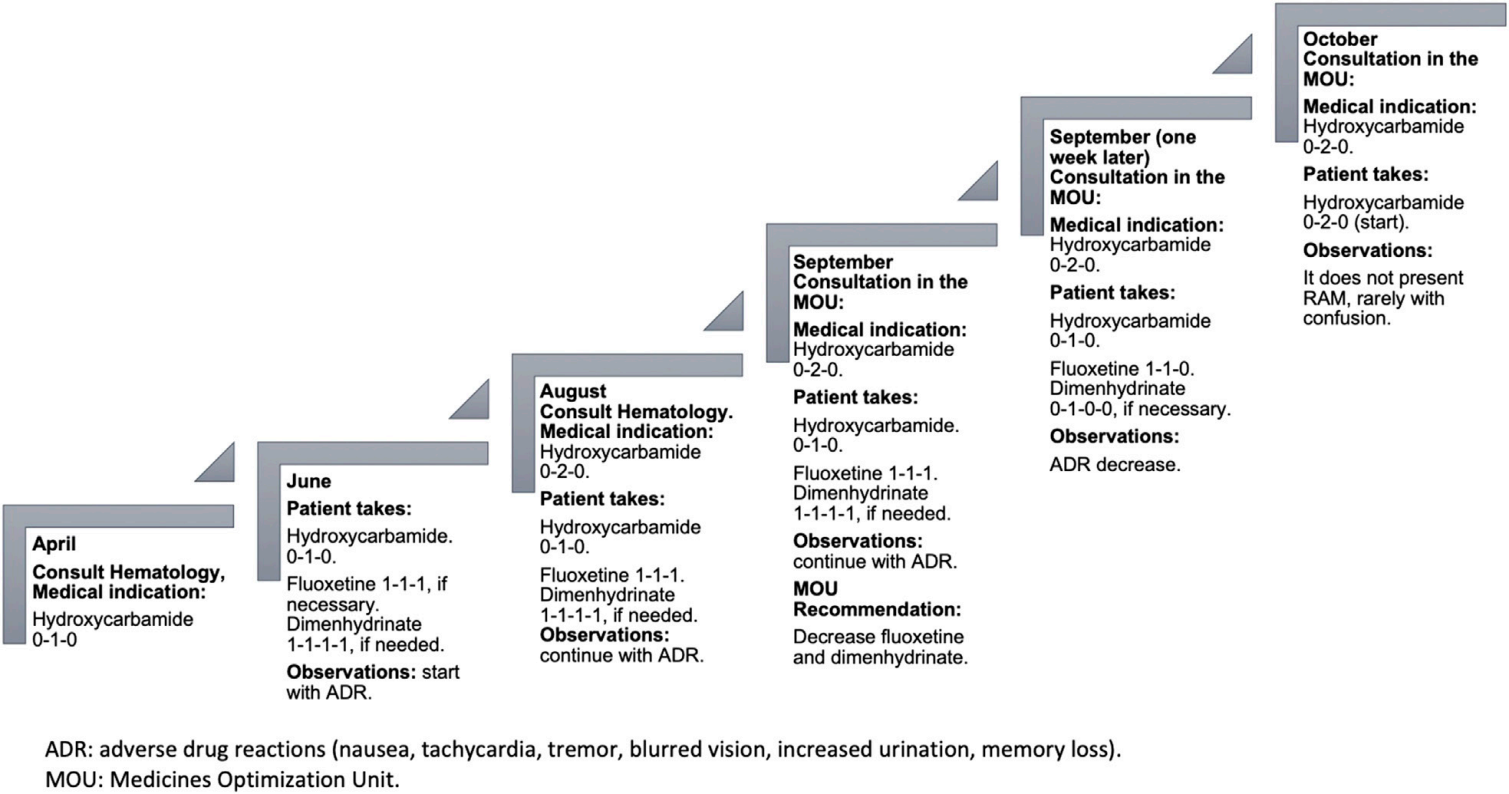
Chronology of the case report.

A week after the recommendations were provided, the patient did not present nausea; he is more active, and tachycardia has decreased along with decreased tremors, less drowsiness, and improved urination. Regarding his memory, he presents confusion sometimes (see Figure 1).

## Discussion

Fluoxetine is a selective serotonin reuptake inhibitor, specifically with a greater affinity for 5HT-2A and 5HT-2C receptors, both related to antidepressant effects, have less norepinephrine reuptake and minimal or no effect on dopamine, melatonin, histamine-I, alpha-I, and cholinergic (Bazire, 2021). One of its main side effects is nausea, which can be increased if used in high doses. In a narrative review on serotonin reuptake inhibitors and adverse effects, they refer to both tachycardia and nausea as adverse reactions (Edinoff et al., 2021). In addition, Sternbach’s criteria for the serotoninergic syndrome also include symptoms of mental confusion, tachycardia, and nausea, which the patient showed (Bazire, 2021). In another case report on the serotoninergic syndrome, a 64-year-old patient used fluoxetine, 25 mg per day, orally and other drugs such as linezolid and metoclopramide, which due to their characteristics also favor the increase in serotonin and symptoms of nausea and tachycardia (Mazhar et al., 2016). In our case, the patient occasionally used 60 mg of fluoxetine per day and manifested nausea and tachycardia.

Another aspect to consider in the presentation of nausea is its interaction with omeprazole (category C) (Lexi-Drugs, 2022), as fluoxetine is a moderate CYP2C19 inhibitor; it increases the effect or toxicity of omeprazole, especially in patients with Zollinger–Ellison syndrome, where high doses of omeprazole are used, given that post-marketing nausea of omeprazole has been reported (Vlase et al., 2010); however, the dose of omeprazole used by the patient in this case was 20 mg per day.

Fluoxetine has a minimal effect on the cholinergic receptor, which contributes to the anti-cholinergic burden also presented by the patient (Bazire, 2021) and interacts with dimenhydrinate (category B), which increased the adverse/toxic effect of the selective reuptake inhibitor of serotonin (Lexi-Drugs, 2022).

Dimenhydrinate is an antiemetic drug and antihistamine of H1 receptors in the gastrointestinal tract, blood vessels, and the respiratory tract. It has a central anti-cholinergic activity. According to the American Geriatrics Society, Beers Criteria (American Geriatrics Society Beers Criteria and Update Expert Panel, 2019), dimenhydrinate is among the group of drugs that should be avoided in the elderly because it is highly anti-cholinergic (according to most anti-cholinergic grading scales) and has potential cardiac effects (Villalba et al., 2013) (Al Rihana et al., 2021). In a longitudinal study on the analysis of the cholinergic burden in drug prescriptions in England from 1990 to 2015, it reflects an increase in the prescription of drugs with a cholinergic burden over the years, and it is the older adults who present a higher cholinergic burden due to the use of these drugs, directly related to the polypharmacy that usually occurs in this population. However, the study did not include over-the-counter medications (Mur et al., 2022). The use of dimenhydrinate in the elderly when they were hospitalized was also recently analyzed, either because they used it previously or initially during hospitalization, despite it being a drug that is not recommended for the elderly, due to its anti-cholinergic property (Cossette et al., 2016).

After analyzing the patient’s perspective, self-medication as a self-care process reflects how people with the ability to influence the patient can make decisions about how to approach the ailments, as indicated by Menéndez (Menéndez, 2009). In this case, the patient and his wife decided to start with fluoxetine, since this medication had helped their son with his mental health. However, the way of use is not adequate, based on the review of the scientific literature. In addition, since the origin of this self-medication is from a new diagnosis (essential thrombosis), an altered state of mind calls “the unmaking of the world” and fluoxetine as a symbol of the reconstitution of the threatened lifeworld (Good, 1994). Finally, in the follow-up appointment, the patient self-perceives an improvement in his health by the reduction of the symptoms and recognizes that self-medication with fluoxetine and dimenhydrinate was harmful.

As limitations of the study, there were no pharmacogenetic tests that could suggest another cause for the increase in the adverse reactions (Micaglio et al., 2021); however, the improvement in the patient’s health condition with the decrease in fluoxetine and dimenhydrinate prevented the increase in complications, which could possibly lead to a medical visit or hospitalization, hand in hand with the economic costs that this would generate for the patient and the system, as indicated by this study by Cutler et al., in their article on annual costs derived from major depressive disorders and the impacts of clinical events (Cutler et al., 2022).

After the process of self-medication, which, as health professionals, we cannot ignore let alone judge, given what has been explained about the damage of cholinergic and serotonergic loads in the elderly and the possibility of generating a syndrome of withdrawal upon the immediate discontinuation of fluoxetine, a gradual withdrawal process was initiated to avoid the exacerbation of symptoms, with the intention of starting with the second hydroxycarbamide tablet and achieving an improvement in essential thrombosis.

## Conclusion

Fluoxetine and dimenhydrinate can increase the serotonergic and cholinergic burden. Their use for self-medication can be beyond the control of professionals. Despite the use of fluoxetine for self-medication, while the tapering off process lasts, this pharmacological therapy must be followed to avoid rebound effects due to the abrupt withdrawal of medications, which makes the situation more complex. The comprehensive evaluation of the patient’s medication in the Medicines Optimization Unit achieved the identification of the problem and minimized the damage, in order to improve his health condition.

## Data Availability

The original contributions presented in the study are included in the article/Supplementary Material; further inquiries can be directed to the corresponding author.
